# Factors Influencing Increased Use of Technology to Communicate With Others During the COVID-19 Pandemic: Cross-sectional Web-Based Survey Study

**DOI:** 10.2196/31251

**Published:** 2022-10-25

**Authors:** Erin Dawe-Lane, Magano Mutepua, Daniel Morris, Clarissa M Odoi, Emma Wilson, Joanne Evans, Vanessa Pinfold, Til Wykes, Sagar Jilka, Sara Simblett

**Affiliations:** 1 Department of Psychology Institute of Psychiatry, Psychology and Neuroscience King's College London London United Kingdom; 2 Biomedical Research Centre South London and Maudsley NHS Foundation Trust London United Kingdom; 3 The McPin Foundation London United Kingdom

**Keywords:** COVID-19, technology use, communication, demographics, digital health, mental health, pandemic, depression, health technology, psychosocial, lockdown, United Kingdom, cross-sectional, survey, social interaction, mental health, social connection, social connectivity

## Abstract

**Background:**

Communication via technology is regarded as an effective way of maintaining social connection and helping individuals to cope with the psychological impact of social distancing measures during a pandemic. However, there is little information about which factors have influenced increased use of technology to communicate with others during lockdowns and whether this has changed over time.

**Objective:**

The aim of this study is to explore which psychosocial factors (eg, mental health and employment) and pandemic-related factors (eg, shielding and time) influenced an increase in communication via technology during the first lockdown in the United Kingdom.

**Methods:**

A cross-sectional, web-based survey was conducted between April and July 2020, examining thoughts, feelings, and behaviors associated with the pandemic, including communicating more using technology (eg, via messaging, phone, or video). We collected sociodemographic information, employment status, mental health service user status, and depression symptoms. We used hierarchical logistic regression to test which factors were associated with communicating more using technology during the lockdown.

**Results:**

Participants (N=1464) were on average 41.07 (SD 14.61) years old, and mostly women (n=1141; 77.9%), White (n=1265; 86.4%), and employed (n=1030; 70.4%). Participants reported a mild level of depression (mean 9.43, SD 7.02), and were communicating more using technology (n=1164; 79.5%). The hierarchical regression indicated that people who were employed and experiencing lower levels of depression were more likely to report increased communication using technology during a lockdown period of the COVID-19 pandemic, and over time, men communicated more using technology. Increased use of technology to communicate was related to greater communication and the inability to see others due to the social distancing measures enacted during the lockdown. It was not related to a general increase in technology use during the lockdown.

**Conclusions:**

Although most participants reported increased use of technology to communicate during a lockdown period of the COVID-19 pandemic, this was more apparent in the employed and those experiencing low levels of depression. Moving forward, we should continue to monitor groups who may have been excluded from the benefits of support and communication using technology.

## Introduction

The COVID-19 pandemic and concomitant restrictions have had an enormous effect on our day-to-day lives [1,2], and technology has been fundamental in enabling us to contact others, access support, and maintain employment throughout periods of lockdown [3]. We wanted to explore whether there was an increase in technological communication during the first COVID-19–related lockdown in the United Kingdom (a period of time when stringent social distancing measures were implemented and people were told to severely limit time spent outside of their own home) and to investigate whether some psychosocial factors (eg, mental health, employment status, and other demographics) and pandemic-related factors (eg, shielding and time) may have contributed to this change.

## Methods

### Design

This cross-sectional study used the results of a web-based survey administered during the United Kingdom’s first national lockdown period of the COVID-19 pandemic with a snowball sampling technique.

### Procedure

The survey was published online between April 24 and June 27, 2020. Participants were presented with an information sheet and asked to provided consent before completing the survey questions.

### Ethics Approval

Ethical approval was received from the King’s College London Research Ethics Committee (HR-19/20-18180).

### Participants

Participants were UK residents aged ≥16 years and were recruited using social media and other web-based platforms (eg, community mental health forums and newsletters) as well as through mental health service user advisory groups.

### Measures

A clinical measure was selected to establish symptoms of depression, but pandemic-specific questions were developed through themes extracted from a series of qualitative interviews with mental health service users and caregivers about coping during the COVID-19 pandemic [4]. Measures of time and demographics were also collected.

#### Clinical Measure

Depression was measured using the patient health questionnaire (PHQ-9) [5], a self-report measure with 9 items corresponding to the Diagnostic and Statistical Manual of Mental Disorders, Fourth Edition diagnostic criteria for major depressive disorder. Scores of 5, 10, 15, and 20 represent the cutoff point for mild, moderate, moderately severe, and severe depression, respectively. We divided participants into severe (>19) and mild to moderate (5-19) groups.

#### Pandemic-Specific Questions

1. Communicating using technology (whether they had been communicating more using technology [eg, messaging, phone, or video] during a lockdown period of the COVID-19 pandemic)
2. Social distancing (whether they were “shielding” and/or “cannot see people I want to see”)
3. Communication (whether they were “talking to people more” and/or “speaking about my problems with someone”)
4. Technology use (had they been “watching TV and films excessively to fill the time” and/or “checking social media and news” and/or “using health and wellness apps”).

#### Time

The survey completion date was subtracted from the date the UK lockdown period of the COVID-19 pandemic started (March 23, 2020).

#### Demographic Characteristics

Participants were queried about their age, gender, ethnicity, employment status, and current mental health service use.

### Data Analysis

A hierarchical logistic regression was performed, using a forced entry method, and included time since the start of the lockdown, age, gender, ethnicity, mental health service user status, level of depression, and employment status (step 1), social distancing impacts (step 2), communication impacts (step 3), and technology use impacts (step 4) of the COVID-19 pandemic lockdown on the dependent variable—increased communication using technology. Interaction terms with “time” were included for all variables to investigate the change at different stages of the COVID-19 pandemic lockdown.

## Results

Participants (N=1464) were on average 41.07 (SD 14.61) years old, and mostly women (n=1141; 77.9%), White (n=1265; 86.4%), and employed (n=1030; 70.4%). Participants reported a mild level of depression (mean 9.43, SD 7.02). Moreover, most participants were communicating more using technology (n=1164; 79.5%) and some (15.1%; n=221) were shielding (Table 1).

Demographic and clinical variables contributed significantly to increased communication using technology and explained 13% of the variance (step 1: *X*^2^_13_=86.25, *P*<.001; Nagelkerke *R*^2^=.13; see Multimedia Appendix 1). People who were employed and had lower levels of depression were more likely to report increased communication via technology. As time in the lockdown period of the COVID-19 pandemic increased, men were more likely to be communicating more using technology. Social distancing (step 2: *X*^2^_4_=25.66, *P*<.001; Nagelkerke *R*^2^=.17) and communication (step 3, *X*^2^_4_=44.78, *P*<.001; Nagelkerke *R*^2^=.23) significantly contributed to the model, explaining an additional 3.7% and 6.3% of the variance, respectively. Those who reported social distancing (“I cannot see the people I want to see”) and/or communication (“I am talking to people more” and “I am speaking openly about my problems with someone”) impacts of lockdown were more likely to be communicating more using technology. Finally, technology impacts of lockdown did not significantly contribute to the model (step 4, *X*^2^_6_=8.53, *P*=.20; Nagelkerke *R*^2^=.24). These results were not affected by removing nonsignificant interaction terms.

**Table 1:** Participant characteristics split by mental health service user status.^a^

Characteristics		Service users (n=285)	Nonservice users (n=1179)	Total (N=1464)	*P* value
Age (years), mean (SD)		36.9 (13.0)	42.1 (14.8)	41.1 (14.6)	<.001
**Gender, n (%)**					.02
	Women	234 (82.1)	907 (76.9)	1141 (77.9)	
	Men	44 (15.4)	259 (22.0)	303 (20.7)	
**Ethnicity, n (%)**					.30
	White	251 (88.1)	1014 (86)	1265 (86.4)	
	Ethnic minorities (excluding White minorities)	30 (10.5)	151 (12.8)	181 (12.4)	
Employed, “yes,” n (%)		156 (54.7)	874 (74.1)	1030 (70.4)	<.001
**Depression severity, n (%)**					<.001
	Minimal	30 (10.5)	349 (29.6)	379 (25.9)	
	Mild	41 (14.4)	318 (27)	359 (24.5)	
	Moderate	59 (20.7)	177 (15)	236 (16.1)	
	Moderately severe	44 (15.4)	103 (8.7)	147 (10)	
	Severe	70 (24.6)	86 (7.3)	156 (10.7)	
Shielding, “yes,” n (%)		66 (23.2)	155 (13.1)	221 (15.1)	<.001
Communicating more using technology, “yes,” n (%)		206 (72.3)	958 (81.3)	1164 (79.5)	<.001

^a^Percentages do not add up to 100% where data were missing.

## Discussion

### Principal Findings

We found that the employed and those experiencing lower levels of depression were more likely to report that they were using technology to communicate more during the first lockdown period of the COVID-19 pandemic and that changes in technology use were motivated by social distancing and communication rather than changes in general technology usage during the first lockdown. Overall, these results indicate that use of technology to communicate with others (eg, to maintain social connection and access support networks) rose independently of any general change in technology use during this period (eg, watching television or checking social media).

During the COVID-19 pandemic, social distancing and self-isolation guidelines meant that people had to shift toward using technology to continue to communicate with others and seek social support. Our results suggest that people experiencing depression were less likely to have adapted to the changes in communication and this was largely anticipated, when one considers that depression can reduce motivation to engage in social interaction [6] and limit social problem-solving abilities [7]. Research has shown that people that have felt more connected to members of their community during the pandemic have experienced lower levels of depression, anxiety, and loneliness [8]. Communication via technology could be an effective coping strategy that individuals employ to maintain social connection, access support, and manage the psychological effects of lockdown [4,9-11]. It is concerning that people experiencing high levels of depression were less likely to report an increased use of technology to communicate with others because this may have left them more vulnerable to the deleterious mental health effects of the lockdown.

Those who were employed during the first COVID-19 lockdown period were more likely to report increased communication using technology. Technology has been an efficacious and convenient tool for communicating with colleagues, working, and accessing vital support networks during the lockdown [12,13]. Social distancing regulations and government advice stipulated that people should work from home wherever possible, and an increase in the prevalence of working from home offers a reasonable explanation as to why those in employment were using technology for communication during the lockdown [13]. Conversely, individuals who were unemployed are likely to have been using technology to communicate with others prior to the COVID-19 lockdown period and to have continued to do so at a similar rate, because they did not face the same pressure to increase their use of technology. Additionally, individuals who are unemployed or from low-income households are more vulnerable to digital exclusion [14] and may have lacked access to the technological resources (eg, data packages, internet access) necessary to increase communication via technology.

The interaction between gender and time is harder to interpret but demonstrates that men were more likely to report a change in their use of technology as the lockdown progressed. This supports evidence that suggests that women found social isolation and distancing more difficult and were more likely to report using internet-based technologies to cope with the stress of the pandemic [10,15]. However, our results suggest that men may experience a delayed response to the impacts of social distancing, turning to technological communication much later in periods of lockdown.

### Strengths and Limitations

Cross-sectional designs cannot determine causality and data from longitudinal studies are needed to disentangle the precise relationship between the different factors examined. The variables we explored may be interrelated; for example, people who reported higher levels of depression may have been less likely to be employed and therefore less likely to report using technology earlier on during the COVID-19 pandemic. Furthermore, withdrawal associated with depression may not be specific to the effects of the COVID-19 pandemic. Although our sample had enough variation to identify specific factors that affect communication via technology, participants needed access to technology and the internet to take part in the web-based survey, which will have introduced sampling bias and likely concealed the most digitally excluded members of society.

### Conclusion

In a climate of unprecedented uncertainty, people have shown incredible resilience and resourcefulness, but have become ever more reliant on technology. Although many people reported that they were communicating more using technology, this change has been most apparent in people who were employed and less prominent in those experiencing higher levels of depression. It is also evident that increased technology use was partly driven by the social distancing and communication consequences of the lockdown rather than a general increase in technology use. As the COVID-19 pandemic continues, we expect that there will be a greater integration of technology into our lives, and therefore we must continue to examine changing technology use and monitor groups who may be excluded from the benefits of support and communication using technology.

## Appendix

Multimedia Appendix 1 Hierarchical logistic regression analysis demonstrating the predictors of communicating more using technology during lockdown.
