# Supplementary material for: Factors Influencing Increased Use of Technology to Communicate With Others During the COVID-19 Pandemic: Cross-sectional Web-Based Survey Study
Source: JMIR Ment Health. 2022 Oct 25;9(10):e31251. doi: 10.2196/31251 (PMC9644246; doi:10.2196/31251)
Supplement: Multimedia Appendix 1 [file mental_v9i10e31251_app1.doc]

|  | | | | | | | | | | | | | | | | | | | | | | | | | | | | | | | | | | | |
| --- | --- | --- | --- | --- | --- | --- | --- | --- | --- | --- | --- | --- | --- | --- | --- | --- | --- | --- | --- | --- | --- | --- | --- | --- | --- | --- | --- | --- | --- | --- | --- | --- | --- | --- | --- |
| *Step 1*  *X^2^ (13) = 86.253, p<.001; Nagelkerke R^2^ =*  *.131)* | | | | | | | | |  | *Step 2*  *X^2^ (4) = 25.656, p<.001; Nagelkerke R^2^ = .168)* | | | | | | | |  | *Step 3*  *X^2^ (4) = 44.777, p<.001; Nagelkerke R^2^ = .231)* | | | | | | | |  | *Step 4*  *X*^2^ (6) = 8.528, p=.202; Nagelkerke R^2^  = .243) | | | | | | | |
|  | *B* | *SE* | *Wald X2* | *df* | *p* | *OR* | *95% CI* | |  | *B* | *SE* | *Wald X2* | *df* | *p* | *OR* | *95% CI* |  |  | *B* | *SE* | *Wald X2* | *df* | *p* | *OR* | *95% CI* |  |  | *B* | *SE* | *Wald X2* | *df* | *p* | *OR* | *95% CI* |  |
| Time | .000 | .014 | .000 | 1 | .991 | 1.000 | .974 | 1.027 |  | .020 | .053 | .138 | 1 | .710 | 1.020 | .919 | 1.131 |  | .026 | .072 | .130 | 1 | .718 | 1.026 | .891 | 1.182 |  | -.018 | .007 | 2.105 | 1 | .147 | .990 | .976 | 1.004 |
| Age | *-.015* | *.007* | *4.821* | *1* | *.028* | *.986* | *.973* | *.998* |  | *-.016* | *.007* | *5.223* | *1* | *.022* | *.985* | *.972* | *.998* |  | -.012 | .007 | 2.842 | 1 | .092 | .988 | .975 | 1.002 |  | -.011 | .007 | 2.105 | 1 | .147 | .990 | .976 | 1.004 |
| Age x time | .000 | .000 | 1.018 | 1 | .313 | 1.000 | .999 | 1.000 |  | .000 | .000 | 1.085 | 1 | .298 | 1.000 | .999 | 1.000 |  | .000 | .000 | .832 | 1 | .362 | 1.000 | .999 | 1.000 |  | .000 | .000 | .916 | 1 | .338 | 1.000 | .999 | 1.000 |
| Ethnicity | -.007 | .317 | .000 | 1 | .983 | .993 | .534 | 1.848 |  | .186 | .331 | .315 | 1 | .575 | 1.204 | .630 | 2.302 |  | .060 | .338 | .032 | 1 | .859 | 1.062 | .548 | 2.059 |  | .023 | .343 | .004 | 1 | .948 | 1.023 | .522 | 2.003 |
| Ethnicity x time | .018 | .019 | .865 | 1 | .352 | 1.018 | .981 | 1.057 |  | .009 | .020 | .192 | 1 | .661 | 1.009 | .971 | 1.048 |  | .007 | .020 | .134 | 1 | .714 | 1.007 | .969 | 1.048 |  | .009 | .020 | .192 | 1 | .661 | 1.009 | .970 | 1.050 |
| Gender | -.098 | .236 | .172 | 1 | .678 | .907 | .571 | 1.441 |  | -.133 | .241 | .306 | 1 | .580 | .875 | .546 | 1.4-3 |  | -.049 | .247 | .040 | 1 | .842 | .952 | .587 | 1.545 |  | -.038 | .249 | .024 | 1 | .878 | .962 | .591 | 1.569 |
| Gender x time | *-.031* | *.015* | *4.430* | *1* | *.035* | *.970* | *.942* | *.998* |  | *-.036* | *.015* | *5.800* | *1* | *.016* | *.964* | *.936* | *.993* |  | *-.033* | *.015* | *4.659* | *1* | *.031* | *.967* | *.939* | *.997* |  | *-.033* | *.016* | *4.574* | *1* | *.032* | *.967* | *.938* | *.997* |
| Mental Health Service use | -.285 | .224 | 1.622 | 1 | .203 | .752 | .485 | 1.166 |  | -.230 | .230 | 1.001 | 1 | .317 | .795 | .507 | 1.247 |  | -.399 | .241 | 2.735 | 1 | .098 | .671 | .418 | 1.077 |  | -.441 | .245 | 3.247 | 1 | .072 | .644 | .399 | 1.039 |
| Mental Health Service use x time | .000 | .014 | .000 | 1 | .991 | 1.000 | .974 | 1.027 |  | -.004 | .014 | .103 | 1 | .748 | .996 | .969 | 1.023 |  | -.007 | .015 | .216 | 1 | .642 | .993 | .965 | 1.022 |  | -.004 | .015 | .064 | 1 | .800 | .996 | .967 | 1.026 |
| Depression | *-.082* | *.013* | *37.659* | *1* | *.000* | *.921* | *.897* | *.946* |  | *-.086* | *.014* | *39.767* | *1* | *.000* | *.918* | *.893* | *.942* |  | *-.062* | *.014* | *18.594* | *1* | *.000* | *.940* | *.914* | *.967* |  | *-.066* | *.015* | *18.944* | *1* | *.000* | *.936* | *.909* | *.965* |
| Depression x time | .000 | .001 | .003 | 1 | .958 | 1.000 | .998 | 1.001 |  | .000 | .001 | .053 | 1 | .817 | 1.000 | .999 | 1.002 |  | .001 | .001 | .355 | 1 | .551 | 1.001 | .983 | 1.030 |  | .001 | .001 | .313 | 1 | .576 | 1.001 | .999 | 1.002 |
| Employment | *.696* | *.195* | *12.689* | *1* | *.000* | *2.005* | *1.367* | *2.940* |  | *.709* | *.202* | *12.349* | *1* | *.000* | *2.033* | *1.369* | *3.020* |  | *.699* | *.206* | *11.496* | *1* | *.001* | *2.012* | *1.343* | *3.015* |  | *.677* | *.209* | *10.501* | *1* | *.001* | *1.968* | *1.307* | *2.964* |
| Employment x time | .006 | .011 | .264 | 1 | .608 | 1.006 | .984 | 1.029 |  | .007 | .012 | .400 | 1 | .527 | 1.007 | .985 | 1.030 |  | .007 | .012 | .302 | 1 | .581 | 1.007 | .983 | 1.030 |  | .005 | .012 | .192 | 1 | .661 | 1.005 | .982 | 1.029 |
| Shielding |  |  |  |  |  |  |  |  |  | -.033 | .247 | .017 | 1 | .895 | .968 | .596 | 1.571 |  | -.071 | .254 | .078 | 1 | .780 | .931 | .566 | 1.533 |  | .011 | .258 | .002 | 1 | .965 | 1.011 | .610 | 1.678 |
| Shielding x time |  |  |  |  |  |  |  |  |  | -.004 | 0.15 | .063 | 1 | .802 | .996 | .967 | 1.026 |  | .000 | .015 | .001 | 1 | .987 | 1.000 | .970 | 1.030 |  | .004 | .016 | .060 | 1 | .807 | 1.004 | .974 | 1.035 |
| I cannot see people I want to see |  |  |  |  |  |  |  |  |  | *1.367* | *.273* | *25.067* | *1* | *.000* | *3.922* | *2.297* | *6.697* |  | *1.490* | *.283* | *27.652* | *1* | *.000* | *4.438* | *2.547* | *7.734* |  | *1.410* | *.287* | *24.127* | *1* | *.000* | *4.098* | *2.334* | *7.194* |
| I cannot see people I want to see x time |  |  |  |  |  |  |  |  |  | .030 | .018 | 2.727 | 1 | .099 | 1.030 | .994 | 1.068 |  | .037 | .019 | 3.802 | 1 | .051 | 1.038 | 1.000 | 1.078 |  | .034 | .020 | 3.013 | 1 | .083 | 1.034 | .996 | 1.075 |
| I am talking to people more |  |  |  |  |  |  |  |  |  |  |  |  |  |  |  |  |  |  | *1.545* | *.332* | *21.615* | *1* | *.000* | *4.686* | *2.443* | *8.986* |  | *1.501* | *.335* | *20.083* | *1* | *.000* | *4.487* | *2.327* | *8.651* |
| I am talking to people more x time |  |  |  |  |  |  |  |  |  |  |  |  |  |  |  |  |  |  | -.009 | .201 | .176 | 1 | .727 | .996 | .972 | 1.020 |  | -.011 | .021 | .277 | 1 | .598 | .989 | .949 | 1.031 |
| I am speaking to others about problems with someone |  |  |  |  |  |  |  |  |  |  |  |  |  |  |  |  |  |  | *.608* | *.201* | *9.113* | *1* | *.003* | *1.837* | *1.238* | *2.726* |  | *.589* | *.204* | *8.309* | *1* | *.004* | *1.802* | *1.207* | *2.688* |
| I am speaking to others about problems with someone x time |  |  |  |  |  |  |  |  |  |  |  |  |  |  |  |  |  |  | -.004 | .012 | .122 | 1 | .727 | .996 | .972 | 1.020 |  | -.005 | .012 | .141 | 1 | .707 | .995 | .971 | 1.020 |
| I watch TV and Film excessively to fill the time |  |  |  |  |  |  |  |  |  |  |  |  |  |  |  |  |  |  |  |  |  |  |  |  |  |  |  | -.007 | .213 | .001 | 1 | .975 | .993 | .654 | 1.508 |
| I watch TV and Film excessively to fill the time x time |  |  |  |  |  |  |  |  |  |  |  |  |  |  |  |  |  |  |  |  |  |  |  |  |  |  |  | -.003 | .012 | .064 | 1 | .800 | .997 | .973 | 1.021 |
| I have been checking social media and news |  |  |  |  |  |  |  |  |  |  |  |  |  |  |  |  |  |  |  |  |  |  |  |  |  |  |  | .334 | .203 | 2.705 | 1 | .100 | 1.396 | .938 | 2.077 |
| I have been checking social media and news x time |  |  |  |  |  |  |  |  |  |  |  |  |  |  |  |  |  |  |  |  |  |  |  |  |  |  |  | .012 | .011 | 1.200 | 1 | .273 | 1.013 | .990 | 1.035 |
| I am using Health and wellness apps |  |  |  |  |  |  |  |  |  |  |  |  |  |  |  |  |  |  |  |  |  |  |  |  |  |  |  | .464 | .254 | 3.349 | 1 | .067 | 1.591 | .968 | 2.616 |
| I am using health and wellness apps x time |  |  |  |  |  |  |  |  |  |  |  |  |  |  |  |  |  |  |  |  |  |  |  |  |  |  |  | .018 | .014 | 1.640 | 1 | .200 | 1.018 | .991 | 1.046 |
| Constant | 1.514 | 2.281 | .441 | 1 | .507 | 4.546 |  |  |  | .853 | 3.193 | .071 | 1 | .789 | 2.348 |  |  |  | -.588 | 4.299 | .019 | 1 | .891 | .555 |  |  |  | 1.832 | 4.804 | .145 | 1 | .703 | 6.243 |  |  |

*Note*. Significant results are presented in italics.
